# Supplementary material for: Mesenchymal Stromal Cells from Perinatal Tissues as an Alternative for Ex Vivo Expansion of Hematopoietic Progenitor and Stem Cells from Umbilical Cord Blood
Source: Int J Mol Sci. 2023 Oct 24;24(21):15544. doi: 10.3390/ijms242115544 (PMC10648510; doi:10.3390/ijms242115544)
Supplement: Supplementary file 1 [file ijms-24-15544-s001.zip › ijms-2629146-supplementary.pdf]

SUPPLEMENTARY FIGURES

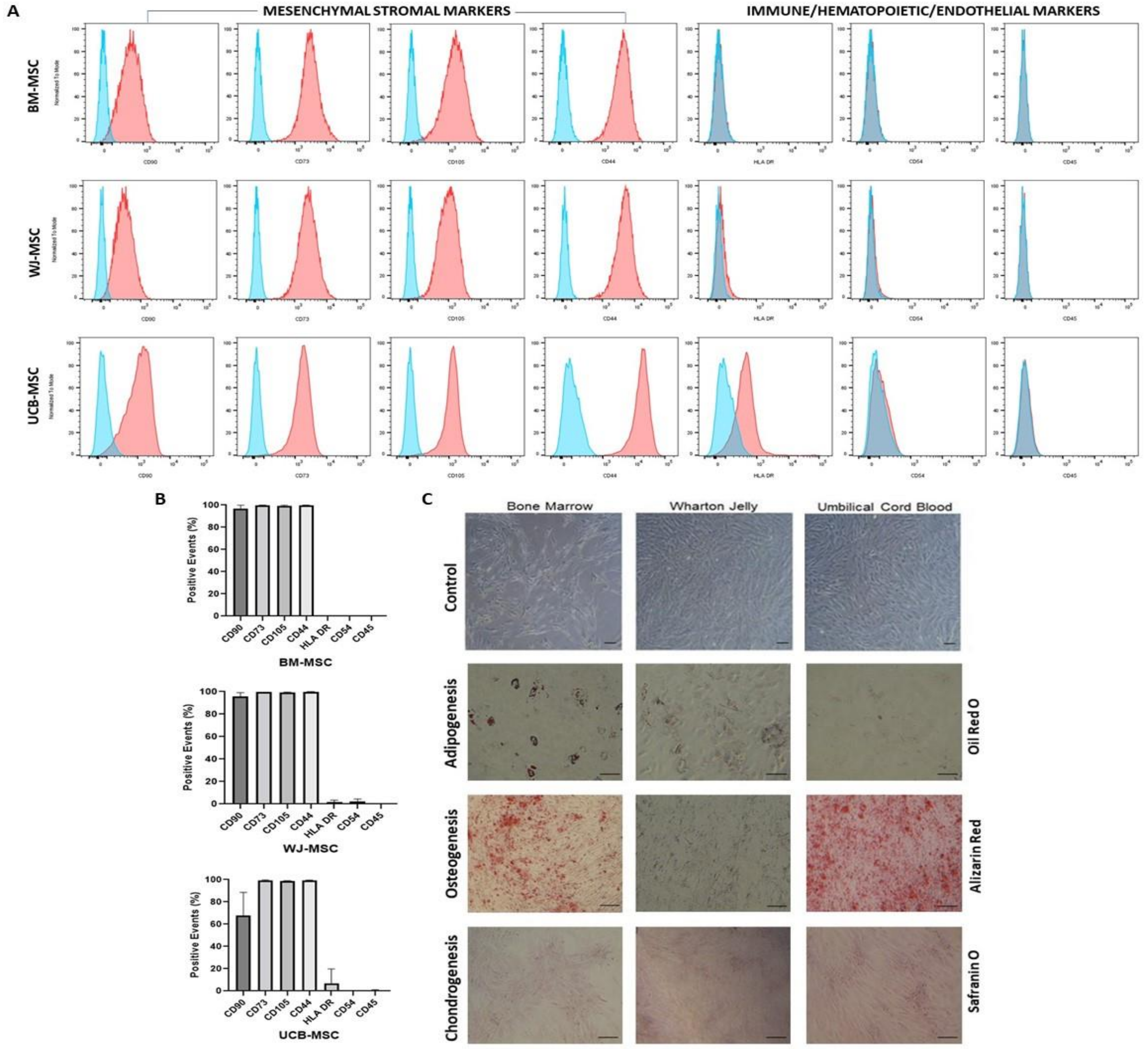

Supplementary Figure S1. Characterization of BM, WJ, and UCB derived Mesenchymal Stromal Cells (MSC). (**A**) Immunophenotypic characterization of the expanded population (passage 3) for the three MSC sources using flow cytometry, employing 7 monoclonal antibodies for positive and negative labeling of the studied population. The figure is representative of the cell lines generated in this study for each MSC source. The blue histograms represent unlabeled cells, the red histograms correspond to the markers evaluated. Debris, doublets, dead cells were removed (**B**) Percentage positive cells to MSC immunophenotypic characterization for the three MSC sources. Data are presented as median with interquartile range from 3 independent experiments with different donors. (**C**) Trilineage differentiation potential of the expanded population (passage 3) for the three MSC sources. BM-MSC, WJ-MSC and UCB-MSC were seeded into adipogenic, chondrogenic, or osteogenic differentiation medium for 14 days, revealing MSC differentiated into adipogenic lineage stained with Oil Red O showing accumulation of lipid vesicles, monolayers chondrogenic differentiation stained with Safranin O showing the change in cell morphology and collagen fibers (rose) and MSC differentiated in osteogenic lineage stained with Alizarin Red showing accumulation of calcium formed. Control showing fibroblast-like morphology and plastic adherence in vitro of MSCs from the three studied sources. Scale bar: 100 μm.

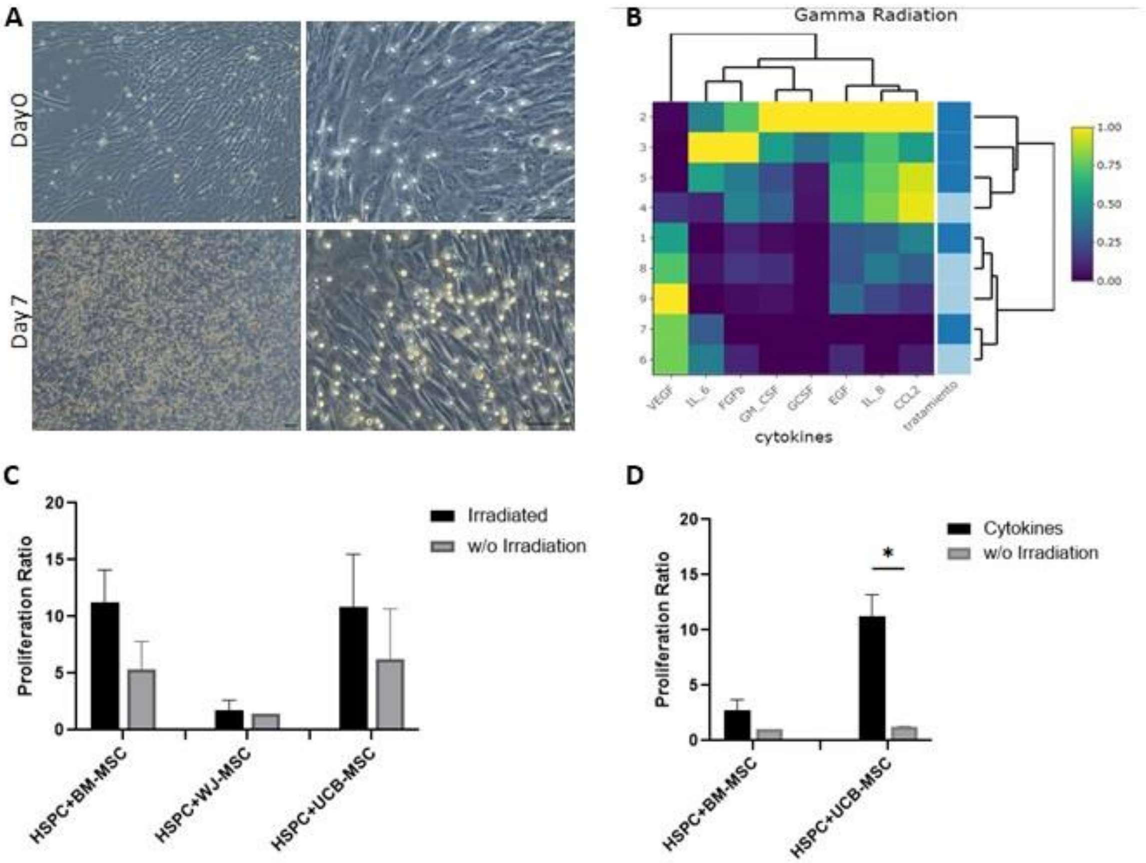

Supplementary Figure S2. **Characterization Definition of Culture Conditions for Hematopoietic Stem Progenitor Cell Expansion**. (**A**) Co-culture observation of HSPCs with WJ-MSCs on the day of seeding fresh HSPCs into the feeder monolayer (day 0) and after 7 days of incubation at 37°C and 5% CO_2 (day 7). The feeder monolayer of WJ-MSCs exhibits fibroblast-like morphology and plastic adherence, and the expansion of HSPCs (round cells in bright yellow color) is observed. Scale bar: 100 µm. (**B**) Heatmap representing the clustering of conditioned media samples from irradiated and non-irradiated MSCs based on their similarity in the secretion of 8 cytokines. Rows represent irradiated samples (dark blue) and non-irradiated samples (light blue). Columns represent the 8 studied cytokines. The color scale corresponds to the relative cytokine secretion, ranging from minimum (0.00) to maximum (1.00) of all values. (**C**) Effect of irradiation of BM-MSCs, UCB-MSCs, and WJ-MSCs on HSPC proliferation in co-culture. Data are expressed as mean ± SEM of 3 independent experiments with different donors of MSCs and HSPCs. (D) Effect of early-acting cytokines (100 ng/mL) on HSPC proliferation in co-culture with BM-MSCs and UCB-MSCs. Results were analyzed using Two-way ANOVA Grouped for two datasets (n=2). *Statistically significant difference ($P \leq 0.05$).

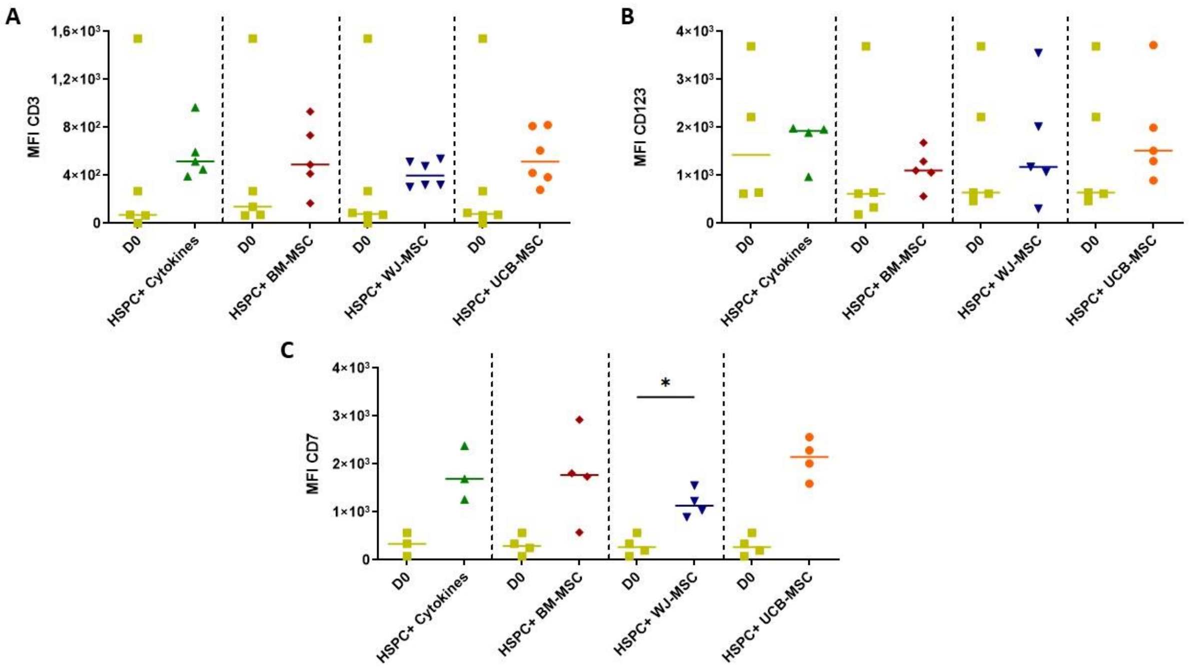

Supplementary Figure S3. Evaluation of Expression of Lineage-Committed Hematopoietic Stem Progenitor Markers (CD123) for Myeloid and (CD3, and CD7) for Lymphoid Lineages of Umbilical Cord Blood Hematopoietic Stem Progenitor Cells (HSPCs) in Co-culture with Mesenchymal Stromal Cells (MSC) (7 day- culture). The expression of lineage-committed progenitor markers on HSPCs cultured with MSCs is assessed using flow cytometry. The expression of these markers is quantified in freshly isolated HSPCs compared to monoculture HSPCs (HSPC+Cytokines, green) treated with early-acting cytokines (SCF, TPO, FLT3). Co-culture with Bone Marrow MSCs (HSPC+BM-MSC, Red). Co-culture with Wharton's Jelly MSCs (HSPC+WJ-MSC, Blue). Co-culture with Umbilical Cord Blood MSCs (HSPC+UCB-MSC, Orange). (**A**) Quantification of MFI for CD3. (**B**) Quantification of MFI for CD123. (**C**) Quantification of MFI for CD7. Statistical analysis by Wilcoxon test in paired data ($p<0.05$), non-significant data.
